# An attenuated African swine fever virus with deletions of the *CD2v* and *A137R* genes offers complete protection against homologous challenge in pigs

**DOI:** 10.1128/jvi.00262-25

**Published:** 2025-09-03

**Authors:** Guorui Peng, Xin Zhao, Xingqi Zou, He Zhang, Junjie Zhao, Xuezhi Zuo, Shuguang Tan, Ruizhi Wu, Xue Guan, Shihua Li, Yuan Xu, Yingju Xia, Xiaoai Xu, Lu Xu, Yuanyuan Zhu, Jinhua Liu, Yebing Liu, George Fu Gao

**Affiliations:** 1 College of Veterinary Medicine, China Agricultural University 630101 , Beijing, China; 2 China/WOAH Reference Laboratory for Classical Swine Fever, China Institute of Veterinary Drug Control 620909, Beijing, China; 3 CAS Key Laboratory of Pathogen Microbiology and Immunology, Institute of Microbiology, Chinese Academy of Sciences 85387, Beijing, China; Lerner Research Institute, Cleveland Clinic, Cleveland, Ohio, USA

**Keywords:** African swine fever virus (ASFV), *CD2v*, *A137R*, double gene knockout strain, attenuation, protection

## Abstract

African swine fever (ASF) has caused a devastating pandemic among domestic and wild swine, leading to significant economic losses in the global swine industry. Recombinant live-attenuated vaccines are a potential option for the control of ASF. However, safe and effective vaccines against the ASF virus (ASFV) are not yet commercially available, and thus, additional vaccine candidates still need to be developed. In this study, we demonstrate that the simultaneous deletion of the ASFV genes *CD2v* and *A137R* from the highly virulent isolate ASFV HuB/HH/2019 substantially attenuated the virulence in swine. All pigs (4/4) infected with 10^5^ TCID_50_ doses of the double gene deletion virus HuB/HH/2019Δ*CD2v*Δ*A137R* (HuBΔ*CD2v*Δ*A137R*) remained healthy during a 27-day observation period, with no fevers (>40.5°C) observed. Cases of viremia were mild and cleared by 24 days post-inoculation (dpi). Antibodies against ASFV p30 were induced, and the inoculated pigs were well-protected against homologous challenges. All inoculated pigs survived after being challenged with 10^2^ HAD_50_ ASFV HuB/HH/2019, and no fever or clinical symptoms developed during the 22-day monitoring period. In contrast, all control group pigs died before 11 dpi (2/2). Cytokine assays and RNA sequencing revealed mild responses elicited by HuBΔ*CD2v*Δ*A137R*. Successful innate and passive immune responses were also triggered. The findings suggest that deletion of the *CD2v* and *A137R* genes can attenuate ASFV, and the deletion mutant virus HuBΔ*CD2v*Δ*A137R* is a promising vaccine candidate.

**IMPORTANCE:**

The emergence of ASF has caused substantial economic losses in the global pig industry. In light of this, the development of a safe and effective vaccine is crucial to control the spread of ASF. In this study, a live-attenuated ASFV strain was developed by simultaneously knocking out the viral genes *CD2v* and *A137R*. The mutant virus exhibited weakened virulence in pigs, elicited robust humoral and cellular immunity, and conferred protection upon challenge with high doses of the parental virus. Notably, no fever or clinical symptoms were observed during a 27-day monitoring period. Consequently, our research presents a highly promising candidate strain for a live-attenuated vaccine, offering a significant step forward in the quest for effective prevention measures. Additionally, the identification of these specific gene targets opens up avenues for further exploration and refinement of strategies aimed at combating ASF outbreaks.

## INTRODUCTION

African swine fever (ASF) is a contagious and lethal hemorrhagic viral disease of domestic and wild swine. The disease requires mandatory declaration to the World Organization for Animal Health (WOAH) (www.woah.org). The disease was first described in Kenya in 1921 (1) and is caused by the ASF virus (ASFV), with mortality rates approaching 100%. Based on the nucleotide sequence of the *B646L* gene encoding the p72 protein, ASFV has been classified into 24 genotypes to date (2, 3). In 2007, a global genotype II ASFV epidemic started in the Caucasus region and spread from Georgia to Russia and Eastern Europe, where it continues to expand regionally in wild boar populations, with occasional outbreaks in pig farms (4). The same ASFV strain reached China and Southeast Asia in 2018, leading to the loss of millions of domestic pigs (5). In 2021, China reported genotype I ASFV strains, the low-virulence and high-virulence variants, and gene deletion of genotype II strains in pig populations (6, 7). In 2023, Zhao et al. reported the isolation of recombinant ASFV from natural genotypes I and II strains (8). The emergence of these strains has posed severe challenges to the prevention and control of ASF. At present, ASF remains widespread globally, causing significant losses to the pig industry. Due to the lack of preventive and therapeutic solutions, ASF is considered a significant global threat.

ASFV is a large double-stranded DNA virus with an approximately 170–193 kilobase (kb) genome encoding 160–175 open reading frames (ORFs) (9). The functions of many of the viral ORFs are unknown, and information regarding their pathogenicity is limited. Only a few ORFs have been described as potential viral factors involved in modulating immune evasion, virus-host interaction, and virulence. Certain genes have been demonstrated to be nonessential for the growth of the virus in macrophages *in vitro*.

As a core component of the icosahedral ASFV virion (10), the A137R protein self-oligomerizes to form a dodecahedron-shaped cage composed of 60 polymers and expressed at the late stages of viral replication (11). The A137R protein of ASFV inhibits type I interferon production via the autophagy-mediated lysosomal degradation of TBK1. Compared with the parental ASFV, ASFV-Δ*A137R* infection significantly increased type I IFN production in porcine alveolar macrophages (PAMs) (12). The anti-pA137R antibodies produced in rabbits or pigs enhanced viral replication of different ASFV strains in primary PAMs. The anti-pA137R antibodies could promote the attachment of ASFV to PAMs and two types of Fc gamma receptors (FcγRs), FcγRII and FcγRIII, mediating the antibody-dependent enhancement (ADE) of ASFV infection. Taken together, these results indicate that anti-pA137R antibodies are able to drive ASFV ADE in PAMs (13). The deletion of the ASFV gene *A137R* from the highly virulent ASFV-Georgia2010 (ASFV-G) isolate induced significant attenuation of virus virulence in swine (14).

The CD2-like protein, also known as CD2v, EP402R, or 8-DR, is responsible for the characteristic rosette formation of erythrocytes around ASFV-infected cells and the binding of extracellular virus particles to erythrocytes; the protein plays a role in the immunomodulation of host responses (15–19). The name CD2v is due to the protein being homologous to the T-cell adhesion molecule CD2 (20). The ASFV CD2v contains each of the domains present in cellular CD2, and thus, it was expected to bind with the CD2 natural ligand CD58/LFA (21). CD2v suppresses type I IFN production and IFN-stimulated gene expression through negatively regulating the cGMP-AMP synthase–STING and IFN signaling pathways (22). Some low-virulence ASFV isolates are non-hemadsorbing and have a truncated or deleted CD2v protein (23, 24).

In this study, we demonstrate that an ASFV strain with concurrent deletions of the *CD2v* and *A137R* genes exhibits complete attenuation in pigs and effectively confers protection against challenge with the virulent parental virus. Consequently, the two-gene-deleted ASFV could potentially be a novel live-attenuated vaccine candidate against ASF.

## RESULTS

### Production of the double deletion mutant HuB△*CD2v*△*A137R*

**Fig 1:**
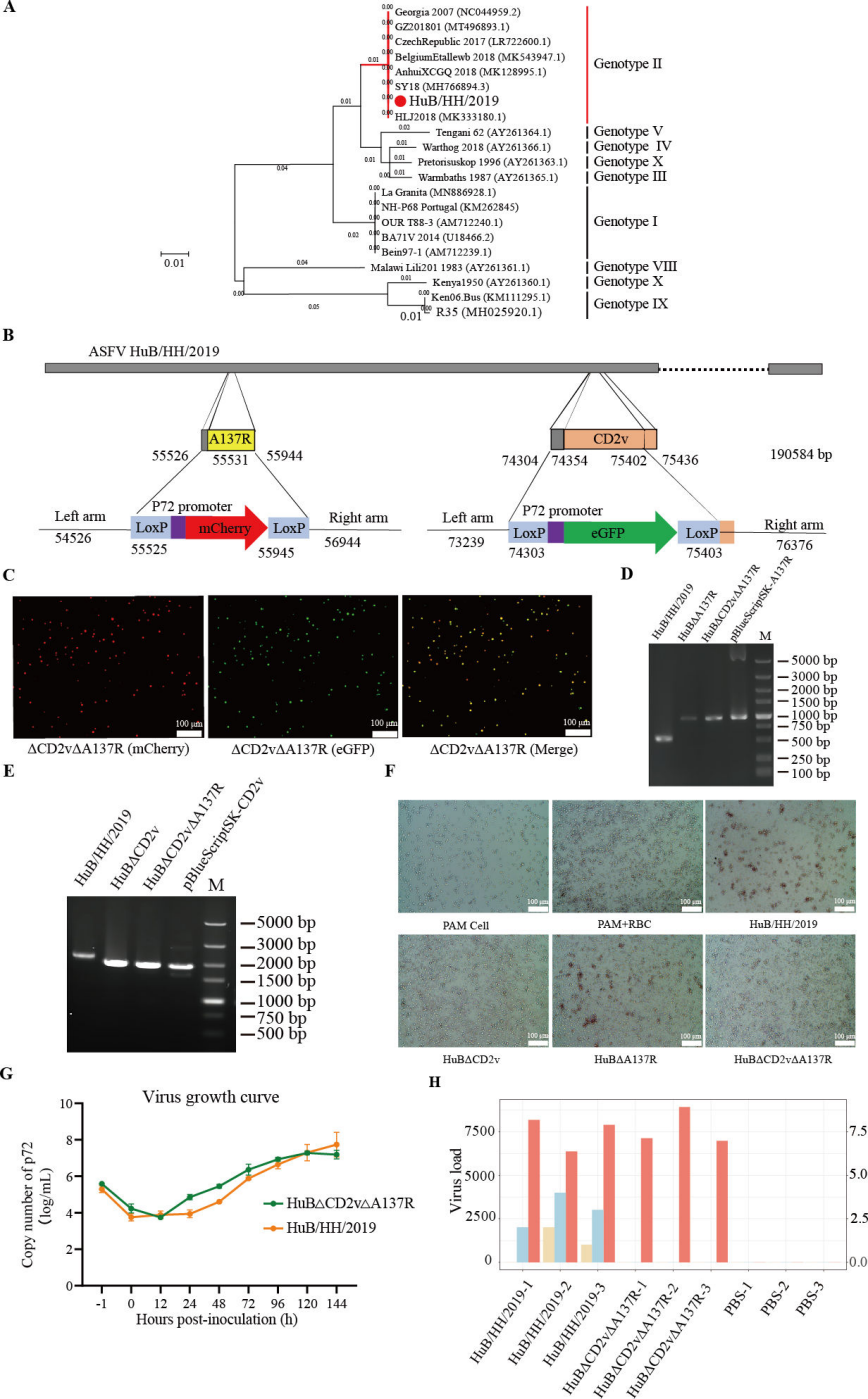
Production and identification of the double deletion mutant *HuB*Δ*CD2v*Δ*A137R.* (**A**) The phylogenetic tree was constructed with 20 ASFV sequences, including HuB/HH/2019, according to the alignment of the *p72* gene. The phylogenetic tree for the *p72* gene was constructed by neighbor-joining. HuB/HH/2019 (red) is a genotype II ASFV strain, and it belongs to the same evolutionary clade as the Georgia 2007 strain prevalent in Russia and Eastern Europe. (**B**) Schematic representation of the double KO strain HuBΔ*CD2v*Δ*A137R*. The genomic segments covering *CD2v* and *A137R* were replaced with the reporter gene cassettes, including *p72-eGFP* and *p72-mCherry*, between two LoxP sites. The numbers indicate the nucleotide positions corresponding to the position on the ASFV HuB/HH/2019 genome. The orange part indicates the *CD2v* gene. The yellow part is the *A137R* gene. (**C**) Fluorescence of mCherry and eGFP in PAMs infected with HuBΔ*CD2v*Δ*A137R* was detected by fluorescence microscopy. PAMs infected with HuBΔ*CD2v*Δ*A137R* were illuminated under green light, and the cells with red fluorescence can be observed (mCherry). PAMs infected with HuBΔ*CD2v*Δ*A137R* were illuminated under red light, and cells with green fluorescence can be observed (eGFP). After the cells were merged, many changed to yellow, indicating that they emitted red and green fluorescence. (**D**) PCR analysis was performed using primers on both sides of *A137R*. Line M is the marker. The WT and KO strains showed bands of different sizes. The double KO strain HuBΔ*CD2v*Δ*A137R* showed only one typical band, indicating the successful KO in the PCR of *A137R*. The *A137R* PCR product sizes were close to the expected PCR sizes; the size of the WT strain was 564 bp, and that of the KO *A137R* strain was 963 bp. (**E**) PCR analysis was performed using primers on both sides of *CD2v*. Line M denotes the marker. The WT and KO strains showed bands of different sizes. The double KO strain HuBΔ*CD2v*Δ*A137R* showed only one typical band, indicating the successful KO of *CD2v*. The *CD2v* PCR product sizes were close to the expected sizes of the WT strain, which was 2,189 bp in size, and the KO *CD2v* strain, which was 1,916 bp in size. The result suggested that the double KO strain (ASFV HuBΔ*CD2v*Δ*A137R*) was successfully constructed and had no contamination by the WT virus. (**F**) Blood cell hemadsorption assays of the WT, the single KO of *A137R* or *CD2v*, and the double KO strains. Both HuBΔ*CD2v* and wild-typ HuBΔ*CD2v*Δ*A137R* lost the capacity for blood cell hemadsorption. (**G**) *In vitro* growth characteristics of the double KO strain and the parental HuB/HH/2019 virus. PAMs were infected with the viruses at an MOI of 0.1. The copy numbers of *p72* genes were detected by real-time qPCR. The virus yields were titrated at the indicated time points post-infection. (**H**) Comparison of the viral load of the double KO strain and the parental HuB/HH/2019 virus via transcriptome analysis. The bar chart illustrates the viral gene loads across different samples. Wheat-colored bars represent *A137R*, blue bars indicate *CD2v* (refer to the right y-axis), and red bars denote the overall viral load (refer to the left y-axis).

As *CD2v* and *A137R* are both related to ASFV virulence, a double -gene knock-out (KO) virus strain was constructed. The KO virus was derived from the ASFV HuB/HH/2019 strain that was isolated from pig specimens by our laboratory and was known to induce acute infection in pigs (25). HuB/HH/2019 belongs to ASFV genotype II, the major pandemic genotype in China and other Asian countries. A phylogenetic analysis showed that HuB/HH/2019 belonged to the same evolutionary clade as the Georgia 2007 strain that is prevalent in Russia and Eastern Europe (Fig. 1A). The protein sequences of CD2v and A137R from HuB/HH/2019 were aligned with those from strains of ASFV genotype II (Georgia 2007, HLJ2018, Belgium Etable wb 2018, Czech Republic 2017, GZ201801, SY18, and Anhui XCGQ), genotype I (BA71V 2014, Bein97, and OUR T88/3), as well as other genotypes (R35 and Ken06.Bus). The results showed that the homology of CD2v was as high as 100% among the seven genotype II strains, compared with 46.8%–58.1% among the genotype I strains and 64.4% among the other two genotypes (Table S1). Meanwhile, the homology of the A137R protein sequence was as high as 100% among the seven genotype II strains, compared with 99.3% among the genotype I strains and 84.7% among the other two genotypes (Fig. 1A; Table S1).

To construct the double KO strain HuB/HH/2019Δ*CD2v*Δ*A137R* (HuBΔ*CD2v*Δ*A137R*), the genomic segments covering *CD2v* and *A137R* were replaced with a reporter gene cassette that included both *p72-eGFP* and *p72-mCherry* by homologous recombination (Fig. 1B). CRISPR/Cas9 was used to increase the recombinant efficiency. The LoxP sites were constructed in the cassette so that we could delete the fluorescent marker to facilitate genome editing of HuBΔ*CD2v*Δ*A137R*. The double KO strain HuBΔ*CD2v*Δ*A137R* was constructed in PAM cells and purified using the limited dilution method with the fluorescent markers eGFP and mCherry (Fig. 1C). HuBΔ*CD2v*Δ*A137R* was passaged and amplified for five cycles in PAM cells and frozen at −80°C for further use.

PCR and RT-qPCR were performed to check the purity of the double KO strain. The PCR analysis was performed using primers on both sides of *A137R*. The wild-type (WT) and KO strains showed bands of different sizes. The double KO strain HuBΔ*CD2v*Δ*A137R* showed a single typical band in the PCR, indicating successful KO of *A137R*. The size of the *A137R* PCR product was near that of the expected WT strain (564 bp), and the size of the KO *A137R* strain was 963 bp (Fig. 1D). The second PCR analysis was performed using primers on both sides of *CD2v*. The WT and KO strains showed bands of different sizes. The double KO strain HuBΔ*CD2v*Δ*A137R* had a single typical band, indicating the successful KO of *CD2v*. The PCR product sizes were close to the expected sizes of the WT strain (2,189 bp) and the KO *CD2v* strain (1,916 bp) (Fig. 1E). The results suggested that the double KO strain (ASFV HuBΔ*CD2v*Δ*A137R*) was successfully constructed and that it was not contaminated with the WT virus. In the whole genome sequencing for the gene-deleted ASFV, we obtained more than 10 Gb of data; of 3.41 × 10^7^ sequences, <5 aligned concordantly with the deleted genes *A137R* or *CD2v*. However, 4.38 × 10^4^ and 3.52 × 10^4^ of the sequences aligned exactly with those of the fluorescent genes *mCherry* and *eGFP*, respectively. The results suggested no contamination with the WT virus, indicating that the HuBΔ*CD2v*Δ*A137R* mutant strain was successfully constructed (Fig. 1D and E; Table S2). In a blood cell hemadsorption assay, compared with the HuB/HH/2019 WT and *A137R* KO strains, both *CD2v* KO and HuBΔ*CD2v*Δ*A137R* had lost the capacity for erythrocyte hemadsorption (Fig. 1F). In this case, the titer of HuBΔ*CD2v*Δ*A137R* was detected by fluorescence and shown as TCID_50_. Real-time quantitative PCR (RT-qPCR) indicated that one TCID_50_ was equal to 94 ASFV *p72* DNA copies for HuBΔ*CD2v*Δ*A137R*, and one HAD_50_ was equal to 1.5 × 10^2^ ASFV *p72* DNA copies for the WT strain HuB/HH/2019. In a virus growth curve assay, there was no significant effect on ASFV growth in PAMs by the *CD2v*/*A137R* double KO strain (Fig. 1G).

Our transcriptome findings demonstrate that the two genes, *CD2v* and *A137R*, were successfully knocked out. Moreover, similar to the virus growth curve assay results, knocking out *CD2v* and *A137R* did not significantly affect the viral load, indicating that their KO has little impact on the amplification of HuB*ΔCD2vΔA137R* on PAM cells (Fig. 1H). The transcriptome analysis results for the host suggest that on PAM cells, the immune response induced by HuBΔ*CD2v*Δ*A137R* is stronger than that induced by HuB/HH/2019. As an immunosuppressive factor, *A137R*, when knocked out, enhances the immune response following viral challenge (Fig. S1). Compared with untreated cells, immune-related pathways were enriched following infection with the KO virus strain HuB*ΔCD2vΔA137R*. It has been suggested that HuB*ΔCD2vΔA137R* infection initiate a series of immune responses, including the induction of inflammatory reactions and the promotion of lymphocyte activation (particularly T cells). Moreover, infection with the HuB*ΔCD2vΔA137R* strain triggers regulatory mechanisms for these immune responses, which respond to pathogen invasion, while maintaining the balance of immune defense and the homeostasis of the internal environment (Fig. S2).

### The virulence of HuBΔ*CD2v*Δ*A137R* was highly attenuated in pigs

**Fig 2:**
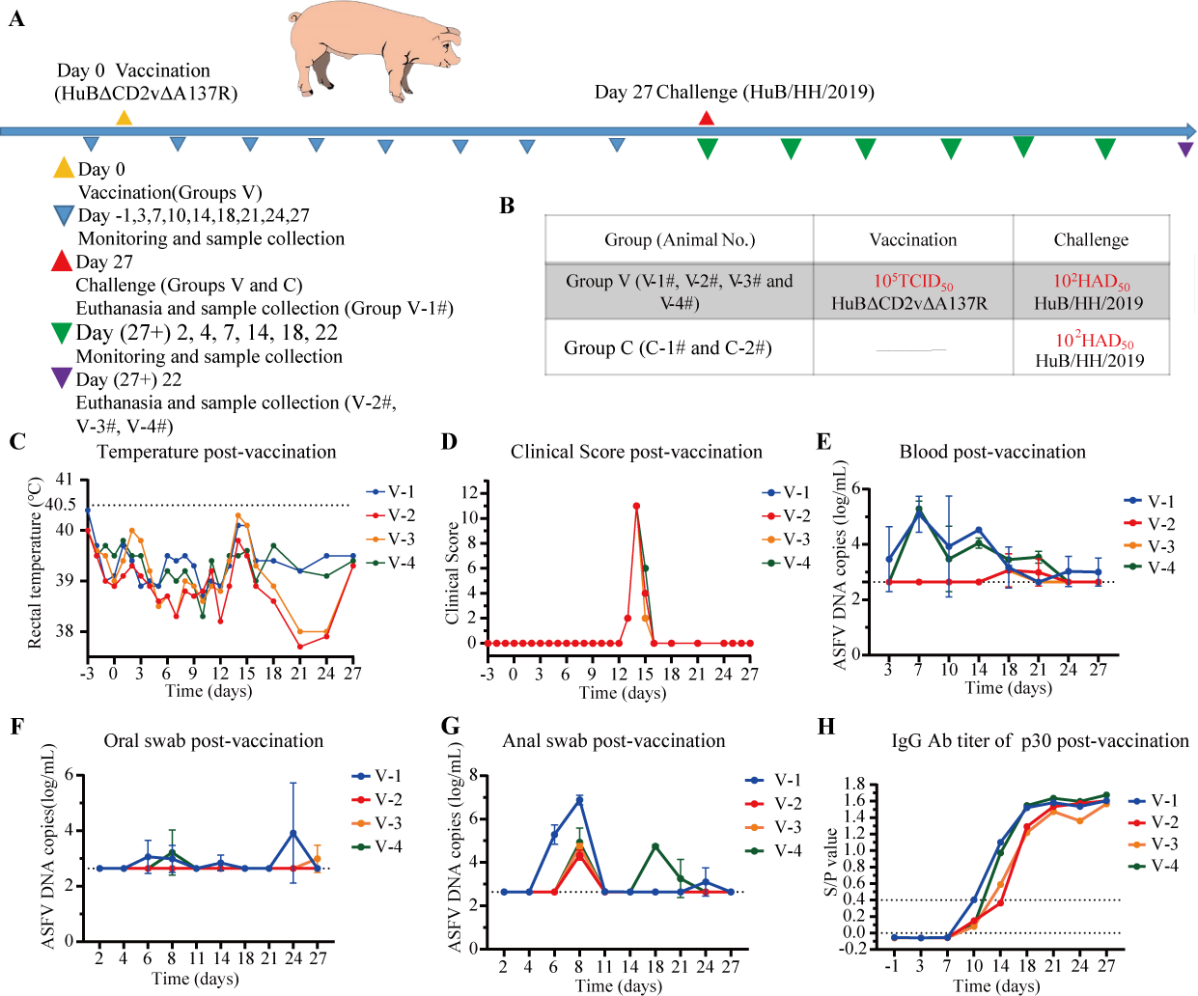
Highly attenuated virulence of HuBΔ*CD2v*Δ*A137R* in pigs. (**A**) Experimental timeline. Animals from Group V were inoculated on Day 0. Pig V-1# from Group V was sacrificed for sample collection at 27 dpi. The pigs V-2#, V-3#, V-4#, C-1#, and C-2# from Groups V and C were challenged by WT HuB/HH/2019 on the same day. The days for double KO virus inoculation, WT ASFV challenge, and sample collection are marked on the timeline using triangles of different colors. (**B**) The table indicates the animal number and the virus titers for inoculation and challenge in each group. (**C**) Rectal temperatures of pigs inoculated with 10^5.0^ TCID_50_ of HuBΔ*CD2v*Δ*A137R,* monitored from Day 3 to Day 27 post-inoculation. (**D**) Clinical scores for pigs inoculated with HuBΔ*CD2v*Δ*A137R* (Group V). (**E**) Viremia in pigs post HuBΔ*CD2v*Δ*A137R* inoculation. The copy numbers of *p72* genes detected by real-time qPCR are shown. The lower limit of detection is 10^3.07^ copies/mL. (**F, G**) Virus shedding in anal and oral swabs from pigs inoculated with HuBΔ*CD2v*Δ*A137R*. The copy numbers of *p72* genes detected by real-time qPCR are shown. (**H**) The antibody levels against the ASFV p30 protein were measured by ELISA in pigs inoculated with HuBΔ*CD2v*Δ*A137R*. Each colored dot represents one piglet. The blue dot is piglet V-1#; the red dot is V-2#; the orange dot is V-3#, and the light green dot is V-4#. The results for each pig at different times are shown.

In our previous work, inoculation with 10 HAD_50_ of HuB/HH/2019 led to the deaths of five out of six piglets, who exhibited typical ASF-related pathological changes (25). In addition, all piglets inoculated with 100 HAD_50_ of HuB/HH/2019 died before 9 dpi (Fig. S3). To investigate the pathogenicity of HuBΔ*CD2v*Δ*A137R*, six 25-day-old pigs (~6.5 kg) were separated into two groups. The four pigs in the first group (group V) were intramuscularly injected with 10^5^ TCID_50_ of HuBΔ*CD2v*Δ*A137R*, and the second group (Group C), which included two pigs, was taken as a control (Fig. 2A and B). The pigs were monitored for 27 dpi, and their body temperatures and clinical symptoms were recorded. All pigs inoculated with HuBΔ*CD2v*Δ*A137R* remained alive during the observation period. Two of the pigs showed a slight increase in body temperature at 14 dpi, but none developed a fever (i.e., above 40.5°C, Fig. 2C). All pigs that received HuBΔ*CD2v*Δ*A137R* showed mild clinical reactions, such as poor appetite and movement, for 2–3 days at around 13–15 dpi. All eventually recovered (Fig. 2D).

Blood samples were collected at 3, 7, 10, 14, 18, 21, 24, and 27 dpi. The load of the double KO virus in the blood was determined by RT-qPCR (Fig. 2E). Notably, the viral load in the blood of the inoculation group was slightly upregulated at 7 dpi and reached the maximum number of copies of 10^5.5^ /mL, but this was reduced to less than 10^4^ after 18 dpi, indicating that HuBΔ*CD2v*Δ*A137R*, the double KO virus, replicated well in the early stage, but it had a shorter viremia period in pigs. Furthermore, we determined the virus titer in oral and anal swabs collected from pigs to check for virus shedding (Fig. 2F and G). Viral loads in the swabs remained at low levels throughout the investigation (Fig. 2F). However, the virus titer in the anal swab increased at 6 dpi and decreased to an undetectable level at 11 dpi (Fig. 2G). The virus in the anal swabs from animal V-4# increased again at 18 dpi, whereas the others maintained a low level throughout the monitoring period (Fig. 2G). The ASFV p30-specific antibody turned positive after 7 dpi and continued to increase. The titer became constant at around 21 dpi (Fig. 2H). This finding indicated that the HuBΔ*CD2v*Δ*A137R* could efficiently induce ASFV-specific antibodies while preserving low virulence in pigs. Therefore, this strain could be an attenuated vaccine candidate.

After the 27-day monitoring period, one pig, V-1# in Group V, was dissected, and the virus titer in each organ was measured (Table S3). The virus was not detected in the liver, lung, spleen, kidney, or duodenum. In the heart, tonsils, and submandibular lymph nodes, the virus in the hepatogastric lymph nodes was at low levels, with the titer below 10^4.7^ copies/mL. This provided evidence of low virus levels remaining in the tissues after inoculation with the double KO strain.

**Fig 3:**
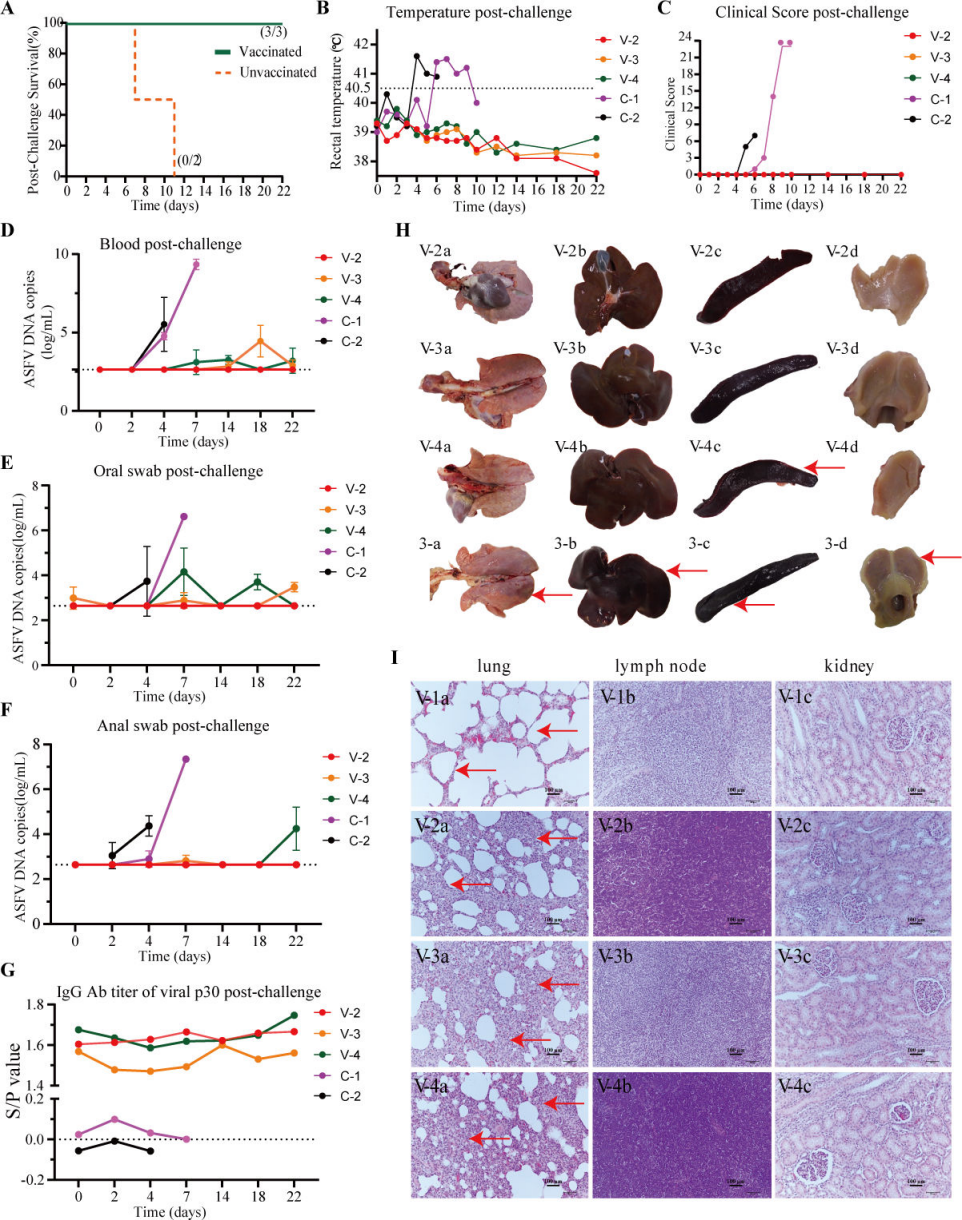
Protection of pigs inoculated with HuBΔ*CD2v*Δ*A137R* after WT ASFV challenge. (**A**) Survival curve of the pigs after challenge with HuB/HH/2019 during the 22-day observation period. (**B**) Rectal temperatures of pigs challenged by 10^2.0^ HAD_50_ of HuB/HH/2019. (**C**) Cumulative clinical scores for pigs after challenge with HuB/HH/2019 (Groups V and C). (**D**) Viremia in pigs after challenge with HuB/HH/2019. The copy numbers of *p72* genes detected by real-time qPCR are shown. The lower limit of detection is 10^3.07^ copies/mL. (**E, F**) Virus shedding in oral and anal swabs from pigs after challenge with HuB/HH/2019. The copy numbers of *p72* genes detected by real-time qPCR are shown. The lower limit of detection is 10^3.07^ copies/mL. (**G**) Antibody levels of pigs from Groups V and C against ASFV p30 protein after challenge with HuB/HH/2019, as measured by ELISA. (**H**) Histomorphologic changes in the lung, liver, spleen, and tonsils of the pigs infected with 10 HAD_50_ of HuB/HH/2019 and euthanized at 9 dpi (3-a, 3-b, 3-c, 3-d) or inoculated with HuBΔ*CD2v*Δ*A137R* and challenged with HuB/HH/2019 (V-2#, V-3#, V-4#). (**I**) Representative microscopic lesions from HuBΔ*CD2v*Δ*A137R* inoculated pigs with (V-2#, V-3#, V-4#) or without (V-1#) WT ASFV HuB/HH/2019 challenge. The blue dot denotes piglet V-1#; the red dot is V-2#; the orange dot is V-3#; the light green dot is V-4#; the purple dot is C-1#, and the black dot is C-2#. The results for each pig at different times are shown.

For pathological investigation, we conducted an analysis of histopathological sections stained with hematoxylin and eosin (HE) from the lungs, lymph nodes, and kidneys of piglet V-1# euthanized on the 27th dpi (Fig. 3I). No severe tissue lesions were observed in the pig, indicating that the HuBΔ*CD2v*Δ*A137R* would be safe for animals. Piglet V-1# showed mild alveolar hemorrhage in the lungs and normal submandibular lymph nodes. In the kidneys, the piglet exhibited no apparent abnormalities in renal tissues.

### HuBΔ*CD2v*Δ*A137R* could protect against ASFV challenge

To determine the protective efficiency of the double KO strain, the three pigs in Group V were challenged with 100 HAD_50_ of the parental strain ASFV HuB/HH/2019 by intramuscular injection at 27 days after the inoculation with HuBΔ*CD2v*Δ*A137R*. The two pigs in Group C that were mock inoculated were also challenged as controls (Fig. 3). All animals challenged were monitored for 22 days, and the survival rates, body temperatures, and clinical symptoms were recorded (Fig. 3A through C). The two pigs that had not received HuBΔ*CD2v*Δ*A137R* inoculation died on Day 7 and Day 11 post-challenge (dpc). However, all three pigs that had received the HuBΔ*CD2v*Δ*A137R* inoculation survived, and none showed ASFV-related symptoms (Fig. 3A and C). The body temperatures of the two pigs in the control group (Group C) were increased at 5 dpc and 7 dpc and remained at a high level for 3–4 days before they died (Fig. 3B). In contrast, the body temperatures of all pigs that received HuBΔ*CD2v*Δ*A137R* before the challenge remained at normal levels throughout the 22 days of observation. Throughout the monitoring period, no symptoms were observed in the pigs that received HuBΔ*CD2v*Δ*A137R*. However, in the control group, clinical signs such as decreased appetite were observed starting from 5 dpc. These symptoms progressively worsened until the pigs eventually died. The pigs in the control group exhibited a range of symptoms, including depression, weight loss, rapid breathing, lying down, redness of the skin, sunken eyes, and loss of appetite (Fig. 3C).

Blood samples of the pigs challenged were collected at 2, 4, 7, 14, 18, and 22 dpc for viremia analysis. In the blood of the control group, high viral loads were observed at 4 dpc, and the levels continued to increase until death. The highest titer levels were 3.3 × 10^5^ or 2.2 × 10^9^ copies/mL (Fig. 3D). In the pigs vaccinated with HuBΔ*CD2v*Δ*A137R*, viral loads were low or undetectable throughout the observation period. Oral and anal swabs were collected from the pigs in both groups (Fig. 3E and F). Regarding the viremia, the virus titer in swabs from the control group was elevated after 2 dpc and continued to increase before death. In the pigs that were inoculated with HuBΔ*CD2v*Δ*A137R*, the virus titer in swabs was less than 1.8 × 10^4^ copies/mL. ASFV was barely detected in blood swabs from the pigs V-2# and V-3# (Fig. 2E; Table 1). Compared with V-2# and V-3#, higher levels of the virus were detected in the swab samples of pig V-4#. To further determine whether these viruses were from the vaccine virus strain or the parental virus strain, more in-depth detection was conducted on pig V-4#, and WT ASFV was found to be present in the blood and swabs from this pig (Table 2). In addition, the viremia and swab virus titers of pig C-1# were much higher than those of pig C-2#. In the control group C, the virus titers of pig C-1# and C-2# were 10^4.7^ and 10^5.5^ copies/mL, respectively, at 4 dpc. The virus titer of C-1# reached 10^9.3^ copies/mL at 7 dpc. Since C-2# died at 7 dpc, the virus titer had not yet reached a high level.

**TABLE 1:** Replication of ASFV in the organs of pigs V-2#, V-3#, and V-4# post-challenge^*a*^

Pig no.	Heart	Liver	Lung	Spleen	Kidney	Duodenum	Tonsil	Submaxillary lymph nodes	Hepatogastric lymph node
V-2#	neg	neg	neg	\	neg	neg	\	neg	neg
V-3#	neg	neg	6.8 × 10^3^	neg	neg	neg	neg	neg	\
V-4#	neg	3.9 × 10^3^	neg	2.1 × 10^5^	neg	neg	5.6 × 10^4^	1.0 × 10^5^	6.6 × 10^3^

^*a*^ The copies/mL of *p72* detected by real-time qPCR are shown. CT values from qPCR greater than 37 or undetectable are labeled as ''neg," whereas the samples not tested are marked as ''\".

**TABLE 2:** Virus detected in the blood and swabs from the pig V-4# post-challenge^*a*^

CT	7 dpc			14 dpc			18 dpc			22 dpc		
	Whole blood	Rectal swab	Oral swab	Whole blood	Rectal swab	Oral swab	Whole blood	Rectal swab	Oral swab	Whole blood	Rectal swab	Oral swab
*p72*	36.66	neg	34.76	neg	neg	neg	neg	neg	32.76	neg	32.46	neg
*A137R*	35.32	neg	35.66	neg	neg	neg	neg	neg	30.99	neg	31.81	neg

^*a*^ The *p72* and *A137R* detected by real-time qPCR are shown. CT values from qPCR greater than 37 or undetectable are labeled as "neg".

The ASFV p30 antibody response in the inoculated pigs remained at a high level but did not increase after the challenge (Fig. 3G), indicating that the HuBΔ*CD2v*Δ*A137R* vaccination provided sufficient protection against the WT virus. It is also possible that the control pigs died before the antibodies were successfully induced.

To examine the efficacy of the HuBΔ*CD2v*Δ*A137R* strain in conferring protection against ASFV infection in porcine hosts, further investigations were conducted, focusing on key organ lesions and pathological sections (Fig. 3H and I). Piglets V-2#, V-3#, and V-4# that received the HuBΔ*CD2v*Δ*A137R* inoculation were euthanized and necropsied on the 22nd dpc. Evaluation of the organs, including the lungs, liver, spleen, and tonsils, revealed no abnormalities in the piglets, except for mild infarction observed in the spleen of piglet V-4# (Fig. 3H). As the pigs in the control group (Group C) had died earlier, we included another control animal in the analysis that had been infected with a lower titer, 10 HAD_50_ of HuB/HH/2019, and this animal was euthanized at 9 dpi. As expected, the organs from this unvaccinated pig exhibited organ lesions. The lungs were congested and enlarged; the liver appeared congested and hemorrhagic with a jelly-like consistency at the edges of the liver lobules; the spleen was enlarged and exhibited infarction; and the tonsils were congested and swollen (Fig. 3H).

During the pathological investigation, we conducted an analysis of histopathological sections stained with HE from the lungs, lymph nodes, and kidneys of piglets V-2#, V-3#, and V-4#, which were euthanized on the 22nd dpc (Fig. 3I). No severe tissue lesions were observed in any of the pigs, indicating that the HuBΔ*CD2v*Δ*A137R* provided effective protection against the corresponding WT ASFV strain. In the lungs, piglets V-2#, V-3#, and V-4# exhibited a slight increase in interstitial hyperplasia in the alveoli. Piglets V-2# and V-4# demonstrated elevated inflammatory cell infiltration in the submandibular lymph nodes, indicating a mild inflammatory effect. No abnormalities were observed in piglet V-3#. None of the piglets exhibited abnormalities in their renal tissues.

Tissues from Group V were also examined to assess residual viral presence. The viral loads in tissues from pigs V-2# and V-3# were very low or undetectable. However, in pig V-4#, the virus could still be detected to some extent in the liver, spleen, tonsils, and lymph nodes. Compared to pigs V-2# and V-3#, higher levels of the virus were detected in the tissue samples of pig V-4#. To further determine whether these viruses were the vaccine or parental virus strain, more in-depth detection was carried out on pig V-4#. The residual viral load in V-4# was identified as the WT ASFV strain (Table 3).

**TABLE 3:** Virus detected in the tissues of pig V-4# post-challenge^*a*^

	Heart	Liver	Lung	Spleen	Kidney	Duodenum	Tonsil	Submaxillary lymph nodes	Hepato-gastric lymph node
*P72*	neg	35.32	neg	29.60	neg	neg	31.51	30.67	34.56
*A137R*	\	neg	neg	29.71	\	\	34.52	33.56	neg
*mCherry*	neg	neg	neg	neg	neg	neg	neg	neg	neg
Cultivation of viruses	\	\	\	Non-fluorescent virus isolated	\	\	Non-fluorescent virus isolated	Non-fluorescent virus isolated	No virus isolated

^*a*^ The *mCherry* and *A137R* detected by real-time qPCR are shown. The virus was isolated and cultured from the real-time qPCR-positive tissue. CT values from qPCR greater than 37 or undetectable are labeled as ''neg," whereas samples not tested are marked as ''\".

### HuBΔ*CD2v*Δ*A137R* activated both innate and adaptive immune responses

**Fig 4:**
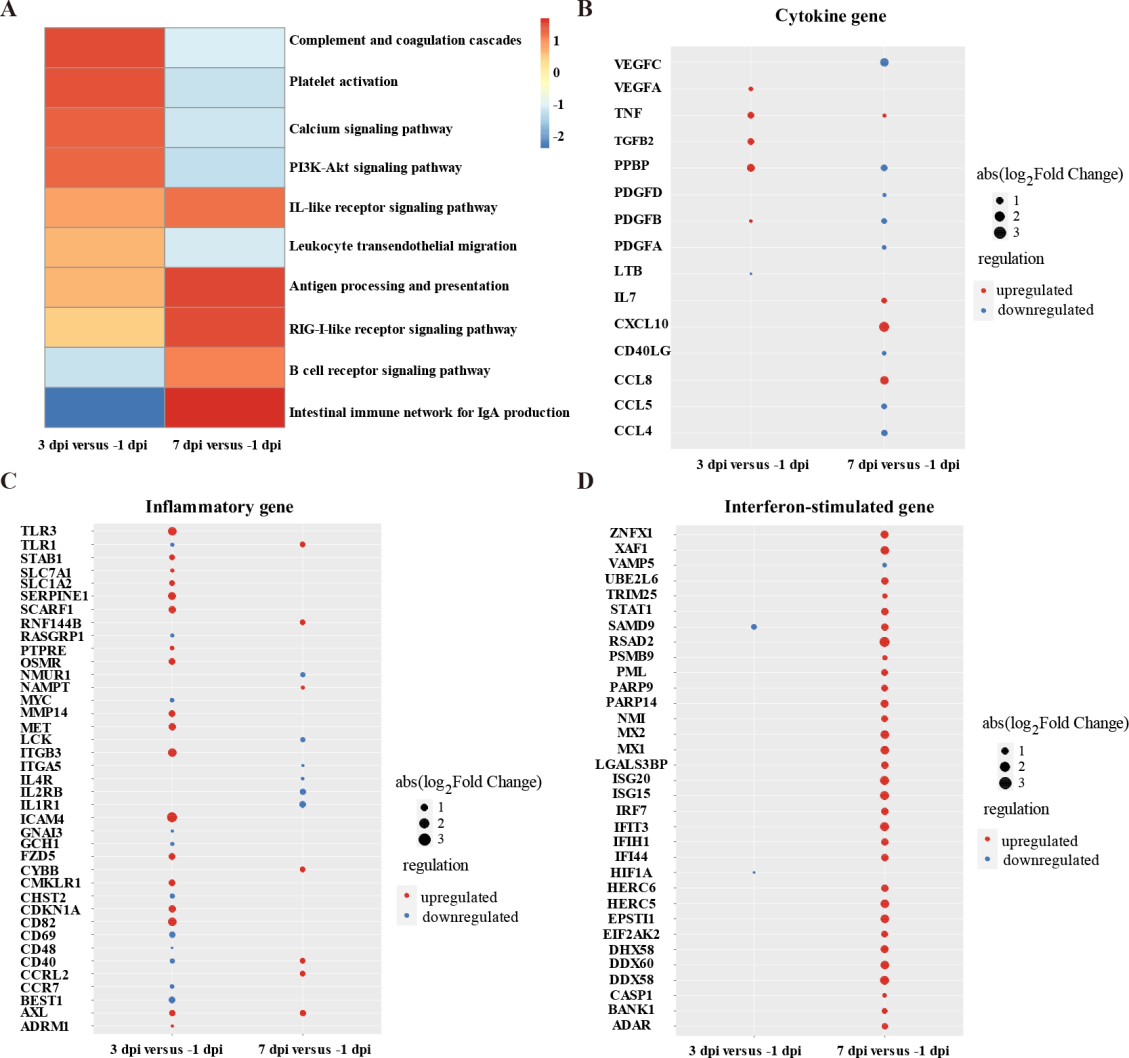
Transcriptome changes in swine after inoculation. (**A**) Heatmap illustrating the results of gene set enrichment analysis (GSEA) based on the KEGG database for genes with *P* values less than 0.1 for host PBMCs at different dpi. The second sample in each set of comparisons serves as the baseline. The color indicates the normalized enrichment score (NES), and the NES of unenriched pathways was set to 0. (B–D) Dot plots showing genes with *P* values less than 0.01 for host PBMCs at different dpi for cytokines (**B**), inflammatory genes (**C**), or interferon-stimulated genes (**D**). Duplicated genes are shown in only one panel. The dot color and size indicate the respective regulation and absolute value of log2-transformed fold change of DEGs compared to the baseline values.

To further analyze the immune response induced by HuBΔ*CD2v*Δ*A137R*, we performed RNA sequencing (RNA-seq) on peripheral blood mononuclear cells (PBMCs) collected from the four pigs in Group V. The samples were collected on Day −1 before vaccination, as well as on Days 3 and 7 after vaccination (Fig. 4). On Day 3 post-inoculation, we observed upregulation of the PI3K-Akt-signaling pathway and leukocyte transendothelial migration, indicating the initiation of the immune response triggered by the attenuated virus. By Day 7, several immune-related pathways were activated. The Toll-like receptor signaling pathway, the RIG-I-like receptor signaling pathway, the antigen processing and presentation pathway, the B cell receptor signaling pathway, and the intestinal immune network for IgA production pathway were all upregulated, suggesting the activation of both innate and adaptive immune responses by HuBΔ*CD2v*Δ*A137R*. These findings were consistent with the cytokine and antibody responses induced by HuBΔ*CD2v*Δ*A137R* and may have contributed to protection against the WT ASFV virus (Fig. 4A). The cytokine responses following HuBΔ*CD2v*Δ*A137R* infection were generally mild. Notably, dysregulation of pivotal inflammatory cytokines such as IL-6, IL-12, IL-18, IFN-α, and IFN-β was not observed post-inoculation with HuBΔ*CD2v*Δ*A137R* (see Fig. 4B and C; Fig. S4). This suggests that the inoculation with the double KO strain did not induce a substantial cytokine storm. Although some inflammatory genes were upregulated at 3 dpi, these returned to normal levels by 7 dpi, indicating that the double KO strain did not significantly disrupt the internal immune homeostasis of the animals (Fig. 4C). Consequently, the pigs exhibited mild or no symptoms after inoculation (Fig. 2). Despite the mild inflammatory reactions, HuBΔ*CD2v*Δ*A137R* successfully activated the interferon pathway, as demonstrated by the significant upregulation of interferon-stimulated genes (ISGs) observed at 7 dpi. Considering the previous findings that both A137R and CD2v negatively regulated the type I interferon pathway, the simultaneous KO of both *CD2v* and *A137R* genes appeared to restore the induction of IFN-related genes (Fig. 4D).

## DISCUSSION

Vaccines play important roles in preventing infectious diseases and in controlling the spread of epidemics (26). The development of a safe and effective ASF vaccine is significant for controlling and eradicating ASFV. However, the lack of fundamental research on the pathogenic mechanisms of ASFV and immune evasion has hindered the development of ASF vaccines and related control technologies. Due to the requirement for both humoral and cellular immune responses for effective immune protection against ASF, attenuated live vaccines may be ideal, and progress has been made by several institutions (27). In 2022, Vietnam approved the commercial use of an attenuated vaccine primarily targeting the deletion of the *I177L* gene (28). The most common approach currently used involves the deletion of one or more virulence-associated genes of ASFV to obtain gene-deficient attenuated strains. This research, focusing on attenuated live ASFV vaccines, seeks to achieve a balance between reducing virulence and maintaining immunogenicity to optimize both safety and effectiveness.

The *A137R* gene KO strain ASFV-G-Δ*A137R,* constructed by knocking out the *A137R* gene in ASFV-G, caused only a transient and mild increase in body temperature in pigs inoculated with 100 HAD_50_, without the typical ASF-related signs. Specifically, in terms of temperature response, three out of five animals had a maximum temperature exceeding 40.5°C, and complete protection could be achieved against the highly virulent parental strain (14).

*CD2v* gene KO has been tested on different ASFV strains as a potential strategy for the development of an attenuated strain. The deletion of *CD2v* from the virulent BA71 genotype I strain led to a complete attenuation upon *in vivo* infection (29), and partial attenuation was observed when *CD2v* was deleted from Benin∆DP148R (30). In contrast, the deletion of *CD2v (8-DR*) in the Malawian strain Lil-20/1 did not alter the onset or course of the disease or the mortality rate, but there was a delay in the onset of viremia (by 2–5 days), and there was a significant reduction in viremia titer (19). Similarly, the deletion of *CD2v* in the Georgia strain did not reduce the virulence *in vivo* but led to a limited reduction in viremia (31). Thus, the deletion of *CD2v* can have variable effects on the virus depending on the ASFV strain used (32). For the genotype II ASFVHLJ/18 strain, the *CD2v*-deficient strain HLJ/18-CD2v-del, when inoculated with 10^3^ TCID_50_ and 10^5^ TCID_50_ in 50-day-old piglets, caused more than half of the animals to develop a fever above 40.5°C for 5–6 days, and deaths occurred in the inoculated animals, indicating that the sole absence of *CD2v* has a relatively limited effect on the virulence of ASFV (33). Furthermore, the deletion of the *CD2v* gene has been identified in several naturally attenuated strains alongside the deletion of other genes (20). The *CD2v* gene has been artificially co-deleted with other viral virulence genes to achieve further attenuation, thereby producing a potential vaccine candidate (20).

Multiple studies have been conducted on gene deletions in ASFV, and the results have indicated that the virulence of several deletion mutants, such as *I73R* (34), *B119L* (35), *I177L* (36), *A137R* (14)*, DP148R* (15)*, EP402R* (29)*, E184L* (37), and *I226R* (38), is markedly decreased in domestic pigs compared to the WT virus. However, in most cases, single gene deletion is not sufficient to construct an attenuated strain that is safe enough to be a vaccine candidate (20). To obtain lower virulence of the mutants, two or more genes are deleted, including *B119L & UK* (39), *EP153R & EP402R* (18)*, A238L & EP402R* (40), *QP509L* & *QP383R* (41), and several successive *MGF* (42) genes.

In this study, the virulence and immunogenicity of the double-gene deletion strain HuBΔ*CD2v*Δ*A137R* (simultaneous deletion of *CD2v* and *A137R*) were validated. Piglets were inoculated with 10^5^ TCID_50_ of HuBΔ*CD2v*Δ*A137R*. During a 27-day observation period, there were slight increases in body temperature to 40.1–40.3°C in two of four piglets on Days 14 and 15, indicating mild clinical symptoms. Serum cytokine analysis and PBMC RNA-seq indicated that the double-gene deletion strain did not induce severe inflammatory reactions in the body, as observed with the virulent strain HuB/HH/2019 (25).

After inoculating the animals with the double-gene deletion strain HuBΔ*CD2v*Δ*A137R*, the virus had a shorter period of viremia compared to the strain ASFV-G-*A137R* that only lacked *A137R* (14). This indicated that the double deletion strain exhibited reduced replication and a lower viral load compared to the *A137R* deletion strain, but that there was still a certain level of virus detected in the tissues at 27 dpi.

In this study, when piglets were challenged with the virulent strain after immunization with HuBΔ*CD2v*Δ*A137R*, the pigs in the control group exhibited typical symptoms of ASF and died; high levels of viremia and viral shedding were observed before death. In contrast, the immunized group did not show any clinical symptoms after challenge; the pigs maintained normal body temperatures, and all piglets survived. This indicates that immunization with HuBΔ*CD2v*Δ*A137R* can effectively reduce virus expansion in pigs. All three immunized animals achieved immune protection and were able to withstand a lethal dose of the virulent strain of ASFV.

In summary, we obtained a double deletion of *CD2v* and *A137R* ASFV mutant virus that exhibited reduced virulence and elicited a robust immune response. The mutant could thus be an attenuated vaccine candidate. Further reduction in virulence could be explored by additional virulent gene deletion; this is currently under investigation in our lab.

## MATERIALS AND METHODS

### Biosafety statement and facility

All experiments with live ASFVs were conducted within the enhanced biosafety level 3 (P3) facilities at the China Institute of Veterinary Drug Control (IVDC) and were approved by the Ministry of Agriculture and Rural Affairs and the China National Accreditation Service for Conformity Assessment (No. CNAS BL0086).

### Cells and viruses

PAMs used in this study were prepared from 38-day-old piglets. Briefly, the intact lung was filled with sterilized washing buffer (RPMI 1640 + 2% penicillin-streptomycin solution (P/S) +2% FBS), and the bronchoalveolar lavage fluid was collected. The cells were collected via centrifugation at 2,000 rpm for 10 min and resuspended in washing buffer. A red blood cell lysis solution was added at a 1:3 ratio (5 mL of cell lysate to 15 mL of lysis buffer), allowed to stand for 5 min, and then centrifuged at 2,000 rpm for 10 min. After removing the erythrocytes and rinsing with washing buffer, the cell pellets were resuspended, and the cells were grown in RPMI 1640 (Gibco, NY, USA) and supplemented with 2% P/S and 8% fetal bovine serum (Gibco, NY, USA). The cells were cultured in an incubator at 37°C under 5% CO_2_.

We performed nucleic acid testing according to the Chinese national standards to ensure that there was no contamination with viruses infecting the swine; this included ASFV, classical swine fever virus (CSFV), and porcine reproductive and respiratory syndrome virus (PRRSV). The ASFV HuB/HH/2019 strain (NMDC accession no. NMDC60214084) that caused acute infection in pigs was isolated in our laboratory from pig specimens. The virus was amplified in primary PAMs and stored at −80°C (25).

### Viral titers

Viral titers of the ASFV strain HuB/HH/2019 in the cell culture medium were estimated by a hemadsorption assay to quantify the endpoint dilution of the ASFV isolate in PAMs by the Reed-Muench method and expressed as 50% hemadsorption doses per milliliter (HAD_50_/mL) per sample. The prepared HuB/HH/2019 titer was 10^8^ HAD_50_/mL, with hemadsorption characteristics. For the gene-deleted strains, the fluorescence emitted directly from *eGFP* and/or *mCherry* was observed. The Reed-Muench method was used to calculate the virus titer, which was expressed as 50% cells emitting fluorescence per mL (TCID_50_/mL) per sample.

### Plasmid design for traditional recombination

The plasmid pBlueScriptSK-2 was used as the backbone (Sangon Biotech, Shanghai, China). The recombination cassette *CD2v* was inserted between the Cla I and EcoR V restriction sites after the T7 promoter. The recombination cassette consists of a left recombination arm that was 1,065 bp upstream of the *CD2v* ORF and identical to HuB/HH/2019 nucleotide positions nt 73239–74303, followed by a LoxP recombination site and the *p72* promoter identical to ASFV HuB/HH/2019 nucleotide positions on the negative strand nt 105559–105597, an enhanced green fluorescent protein (*eGFP*), a second LoxP recombination site, and a right recombination arm 974 bp downstream of *CD2v* identical to HuB/HH/2019 nucleotide positions nt 75403–76376. When knocking out the *CD2v* gene, we retained 33 bp of the *CD2v* gene, from nt 75403–75436 in the HuB/HH/2019 genome.

The recombination cassette *A137R* was inserted between the Hind III and EcoR V restriction sites after the T7 promoter in pBlueScriptSK-2. The recombination cassette consists of a left recombination arm 1,000 bp upstream of the *A137R* ORF and identical to ASFV HuB/HH/2019 nucleotide positions nt 54526–55525, followed by a LoxP recombination site and the *p72* promoter identical to ASFV HuB/HH/2019 nucleotide positions on the negative strand nt 105559–105597, mCherry Fluorescent Protein (mCherry), a second LoxP recombination site, and a right recombination arm 1,000 bp downstream of *A137R* and identical to HuB/HH/2019 nucleotide positions nt 55945–56944.

### Construction of CRISPR/Cas9 expression plasmids

Six DNA fragments encoding gRNA aligned to six selected regions within the *CD2v* and *A137R* ASFV genes (three target sites per gene) were designed (Table S4). The target sequences were unique in comparison to the rest of the genome and were located adjacent to a 3-nt-long Protospacer Adjacent Motif sequence serving as a binding signal for Cas9 nuclease. The pX458 vector was obtained by cloning the designed gRNA encoding oligonucleotides into a vector backbone (Genscript, Nanjing, China). The target sequences, including the Protospacer Adjacent Motif and their positions, are listed in Table S4.

The plasmid encoded an endonuclease Cas9 (hSpCsn1) lacking two NLS and an antibiotic resistance gene (ampicillin) to facilitate the selection of positive clones. The sequence encoding gRNA and CRISPR RNA was encoded under the control of a U6 promoter. *Escherichia coli* (*E. coli*) G10 cells were transformed with the obtained construct and provided by Genscript as bacterial glycerol stocks. *E. coli* was cultivated overnight in LB medium supplemented with ampicillin, then sedimented by centrifugation, and subjected to plasmid DNA extraction using an EndoFree Maxi Plasmid Kit (TIANGEN, Beijing, China), according to the manufacturer’s protocol. The plasmids were used for further transfection of selected cells.

### Construction of gene-deleted HuBΔ*CD2v*Δ*A137R*

The *CD2v* and *A137R* genes were deleted from the ASFV HuB/HH/2019 genome and replaced with *eGFP* and *mCherry* genes by homologous recombination between the ASFV HuB/HH/2019 virus and a recombinant plasmid comprising the *eGFP* expression cassette under the control of the *p72* promoter and two sequences of about 1,000 bp flanking the *CD2v* and *mCherry* genes. The template plasmids and one of three CRISPR/Cas9 expression plasmids (approximately 1 µg) were transfected into PAMs in a 24-well plate using Lipofectamine 3000 Reagent (Thermo Fisher, Waltham, MA, USA). After 24 h, the transfected cells were infected with ASFV HuB/HH/2019 at a multiplicity of infection (MOI) of 3 and the cells were then further cultured at 37°C under 5% CO_2_. The cells exhibiting fluorescence were picked out under a fluorescence microscope (Olympus, Tokyo, Japan) and further screened. The recombinant virus was purified by limiting dilution. The KOs of *A137R* and *CD2v* were constructed separately. The double KO strain HuBΔ*CD2v*Δ*A137R* was produced by KO of *CD2v* based on HuBΔ*A137R*. The final recombinant virus was amplified in PAMs. The purity of the recombinant virus was determined by PCR and RT-qPCR. The PCR forward and reverse primers were designed based on the sequences in the left and the right flanking regions, respectively (Table S5). RT-qPCR forward and reverse primers and Internal Oligo primers were designed based on the sequences in the ORF region using Primer Premier 3 software (https://primer3.ut.ee/, Table S5). Recombinant viral DNA was extracted and next-generation sequenced.

### Hemadsorption test

The PAMs were cultured in 96-well plates. ASFV was incubated, and porcine erythrocytes were added to the PAM cell plates. The cells were incubated at 37°C in an atmosphere of 5% CO_2_ for 7 days, and the presence of erythrocytes absorbed on the surface of the PAMs was checked every day using a microscope.

### Kinetic curve of HuBΔ*CD2v*Δ*A137R*

RT-qPCR targeting the ASFV *p72* gene was employed to detect the proliferation and titers of the recombinant and parental strains of HuB/HH/2019 after they were inoculated at an MOI of 0.1 in PAMs. Samples were taken at 12, 24, 48, 96, 120, and 144 h to measure the copy number of the viral *p72* gene.

### mRNA sequencing on PAMs

PAMs (3 × 10⁶ per well) were seeded in 6-well plates and infected with ASFV strains HuB/HH/2019 and HuBΔCD2vΔA137R at an MOI of 0.01. After 1 h of incubation, the virus-containing medium was removed, the cells were washed twice with PBS, and the fresh medium was added. At the indicated time point (48 hpi), 500 µL of Trizol was added to each well; cells from two wells were combined into one sample through repeated pipetting and mixing, transferred to a cryopreservation tube, snap-frozen in liquid nitrogen, and stored at -80°C for subsequent transcriptome sequencing. Messenger RNA was purified from total RNA using poly-T oligo-attached magnetic beads. After fragmentation, the first-strand cDNA was synthesized using random hexamer primers, followed by the second-strand cDNA synthesis. The library was ready after end repair, A-tailing, adapter ligation, size selection, amplification, and purification, at which point it was checked with Qubit and real-time PCR for quantification and a bioanalyzer for size distribution detection.

### Animal experiments

All animal experiments were approved by the Animal Welfare and Ethics Committee of IVDC (approval number IVDC-FU-2022-00246), and the ASFV cultivation and animal experiments were conducted in an animal biosafety level 3 (ABSL-3) laboratory. In the artificial infection experiment, six piglets were inoculated with HuB/HH/2019 with 10 and 100 HAD_50_. To evaluate the effect of the *CD2v* and *A137R* gene deletions on the virulence of ASFV HuB/HH/2019 *in vivo*, four Landrace pigs free of both ASFV antigen and antibody were inoculated intramuscularly (i.m.) with 10^5^ TCID_50_ of HuBΔ*CD2v*Δ*A137R*. Two pigs were set as the controls. The rectal temperatures were measured every day. Oral and anal swabs and blood samples were collected for viral nucleic acid detection via probe-based RT-qPCR or antibody assays by an ELISA. Clinical symptoms such as high fever, inappetence, depression, diarrhea, waddling, reluctance to stand, skin cyanosis, and arthrocele were observed and recorded daily throughout the experiment. The table for the clinical scoring system is provided in Table S6.

On Day 27 post-inoculation, the pig V- 1# from Group V was euthanized using zoletil50 (V.I.C. Pet Care, France) at the end of the observation period, and the tissues, including heart, liver, lung, spleen, kidney, duodenum, tonsil, submaxillary lymph nodes, and hepatogastric lymph node, were collected for viral load assessment via RT-qPCR. The pigs from Group V were inoculated with 10^5^ TCID_50_ of HuBΔ*CD2v*Δ*A137R,* and those from Group C were challenged by intramuscular injection with 10^2^ HAD_50_ of HuB/HH/2019. Clinical observation, sampling, and detection were performed as described above.

On Day 22 post-challenge, the pigs were necropsied and inspected for gross tissue lesions, in the lung, liver, spleen, and tonsils. The tissues of the heart, liver, lungs, spleen, kidneys, duodenum, tonsils, submaxillary lymph nodes, hepatogastric lymph nodes, and hilar lymph nodes were collected for the detection of ASFV nucleic acids via RT-qPCR. The lung, lymph node, and kidney tissues were fixed in paraformaldehyde, sectioned, and HE stained for histopathological analysis. The tissues from piglets inoculated with HuB/HH/2019 at 10 HAD_50_ and euthanized on Day 9 after infection were used as controls in the histopathological analysis.

### Quantification of the ASFV load

The ASFV was diluted to achieve specific HAD_50_ or TCID_50_ values, and the *p72* gene was detected by RT-qPCR. Probe-based RT-qPCR targeting the ASFV *p72* gene was performed to determine the ASFV load in the blood and tissues. The numbers of virus copies were calculated based on the RT-qPCR CT values and the copies of the *p72* gene from the positive plasmid standard curve (43). The tissues were cut into soybean-sized pieces and placed in sterile tubes containing steel beads. Then, 1,000 µL PBS was added, after which the tissue was placed in a homogenizer at 400 strokes per minute for 30 s, and this was repeated three times with a 10 s interval between each cycle. The homogenate was then centrifuged at 4°C, 5,000 rpm for 10 min. The supernatant was collected and transferred to another sterile microcentrifuge tube and labeled for further use. In brief, DNA was extracted from each sample using an animal virus DNA/RNA kit (TIANLONG, Shanxi, China). RT-qPCR was performed using Premix Ex Taq (probe qPCR) reagent (Sangon Biotech, Shanghai, China). The primers and probes are listed in Table S5. Copy numbers of the ASFV genome samples were calculated according to the standard curve established by the detection of the standard plasmid containing the ASFV HuB/HH/2019 gene pUC57-B646L (Sangon Biotech, Shanghai, China). The standard curve of the ASFV *p72* gene in the quantitative polymerase chain reaction was plotted based on the equation y = −3.2837x + 41.108, where y is the CT value and x is the number of copies of the *p72* gene. The limit of detection for the RT-qPCR assay was 10^3.07^ copies/mL. We classified the virus copies as follows: levels below 10^5^ copies per milliliter were considered low, those ranging from 10^5^ to 10^7^ copies per milliliter were regarded as moderate, and concentrations exceeding 10^7^ copies per milliliter were categorized as high.

### Indirect ELISA

The ASFV-specific antibodies targeting p30 were assayed by the indirect ELISA method using an ASFV antibody ELISA kit (Jinnuo Zhenduan, Beijing, China) following the manufacturer’s protocol. Briefly, the serum sample, positive control, and negative control were added to the ELISA plates and incubated for 30 min at room temperature (RT). Horseradish peroxidase (HRP)-labeled anti-pig IgG, used as the secondary antibody, was incubated for 30 min at RT. The chromogenic reaction began with the addition of 3,3',5,5'-tetramethylbenzidine (TMB) substrate and ended with a stop buffer. The optical density at 450 nm (OD_450_) values were read using an auto ELISA detector (BioTek Instruments, Winooski, VT, USA). An S/*P* value ≥ 0.4 is considered positive.

### mRNA sequencing on PBMCs

We performed RNA-seq on PBMCs collected from the four pigs in Group V. The samples were collected on Day −1 before vaccination, as well as on Days 3 and 7 after vaccination. The total RNA was isolated using the Invitrogen TRIZOL Reagent (Carlsbad, CA, USA), after which the concentration and integrity of the sample RNA were determined using a NanoPhotometer (IMPLEN, CA, USA) and an Agilent 2100 RNA nano 6000 assay kit (Agilent Technologies, CA, USA), respectively. Then, rRNA was depleted using a KAPA RiboErase Kit Human/Mouse/Rat (KK8482), and a total of 1-3 µg of RNA per sample was used as input material for the RNA sample preparation. Sequencing libraries were generated using a VAHTS Universal V6 RNA-seq Library Prep Kit for Illumina (NR604-01/02) following the manufacturer’s recommendations, except that the RNA sample was not purified using poly-T oligo-attached magnetic beads, but rather directly broken into short fragments. Index codes were added to attribute sequences for each sample. The cluster generation and sequencing were performed on a NovaSeq 6000 S4 platform using a NovaSeq 6000 S4 Reagent kit V1.5.

### Bulk RNA-seq data processing and statistical analysis

Raw RNA-seq reads were trimmed, and reads with low-quality bases were discarded using trimmomatic (version: 0.39) (44). Clean reads were mapped against a manually combined reference genome of the Ensembl *Sus scrofa* genome (*Sus_scrofa.Sscrofa11.1.dna_sm.toplevel.fa*) and the ASFV genome (*MH6894_4.fa*) with a manually combined annotation file comprising the Ensembl *Sus scrofa* GTF file (*Sus_scrofa.Sscrofa11.1.94.gtf*) and the ASFV GTF file (*MH766894_2.gtf*) using STAR (version: 2.7.10b). The resulting Bam file after mapping was imported into R Studio (version: 4.3.1), and the raw counts of each mRNA were quantified using Rsubread (version: 2.14.2) (45).

Differentially expressed genes (DEGs) were identified with DESeq2 (version: 1.40.2) (46), and genes with a log2-transformed fold change greater than 1 and a *P* value less than 0.05 were designated as DEGs. Human homologs of genes with *P* values less than 0.05 and their ENTREZID were obtained using org.Hs.eg.db (version: 3.17.0). Gene set enrichment analysis (GSEA) was performed with the "gseKEGG" function in the R package clusterProfiler (version: 4.8.3) (47).

Statistical significance was determined using the Holm-Sidak test, and a *P*-value of 0.05 was considered statistically significant. Similar results were obtained from three independent experiments. Statistically significant differences between groups were analyzed using GraphPad Prism 8.0 software (GraphPad, La Jolla, CA, USA).

## Data Availability

The transcriptome sequencing data that support the findings of this study are available at the National Genomics Data Center (NGDC) of China National Center for Bioinformation (CNCB) with the accession ID PRJCA025619.

## SUPPLEMENTAL MATERIAL

Supplemental material jvi.00262-25-s0001.docx Figures S1 to S4; Tables S1 to S6.

ASM does not own the copyrights to Supplemental Material that may be linked to, or accessed through, an article. The authors have granted ASM a non-exclusive, world-wide license to publish the Supplemental Material files. Please contact the corresponding author directly for reuse.
