# Supplementary material for: An attenuated African swine fever virus with deletions of the CD2v and A137R genes offers complete protection against homologous challenge in pigs
Source: J Virol. 2025 Sep 3;99(9):e00262-25. doi: 10.1128/jvi.00262-25 (PMC12456006; doi:10.1128/jvi.00262-25)
Supplement: Supplemental material — Figures S1 to S4; Tables S1 to S6. [file jvi.00262-25-s0001.docx]

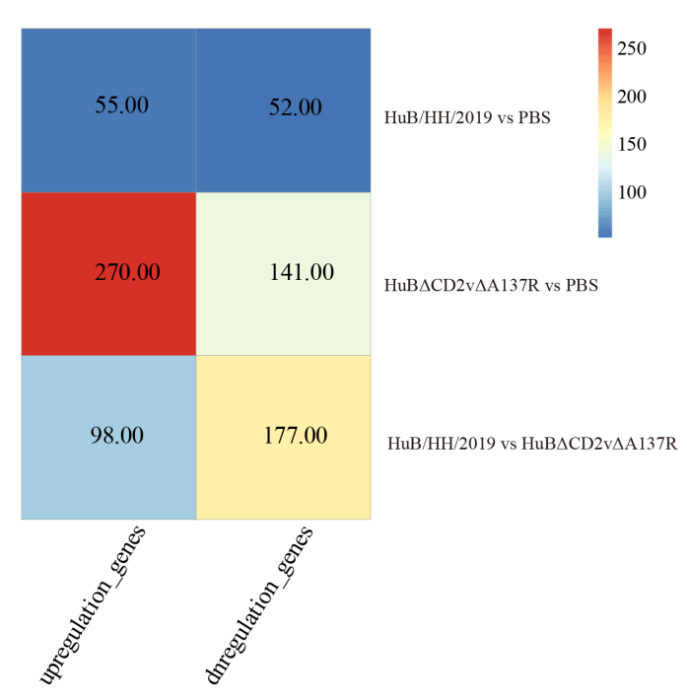


Fig S1. This heatmap displays the number of differentially expressed genes (DEGs) identified across comparisons between different groups.


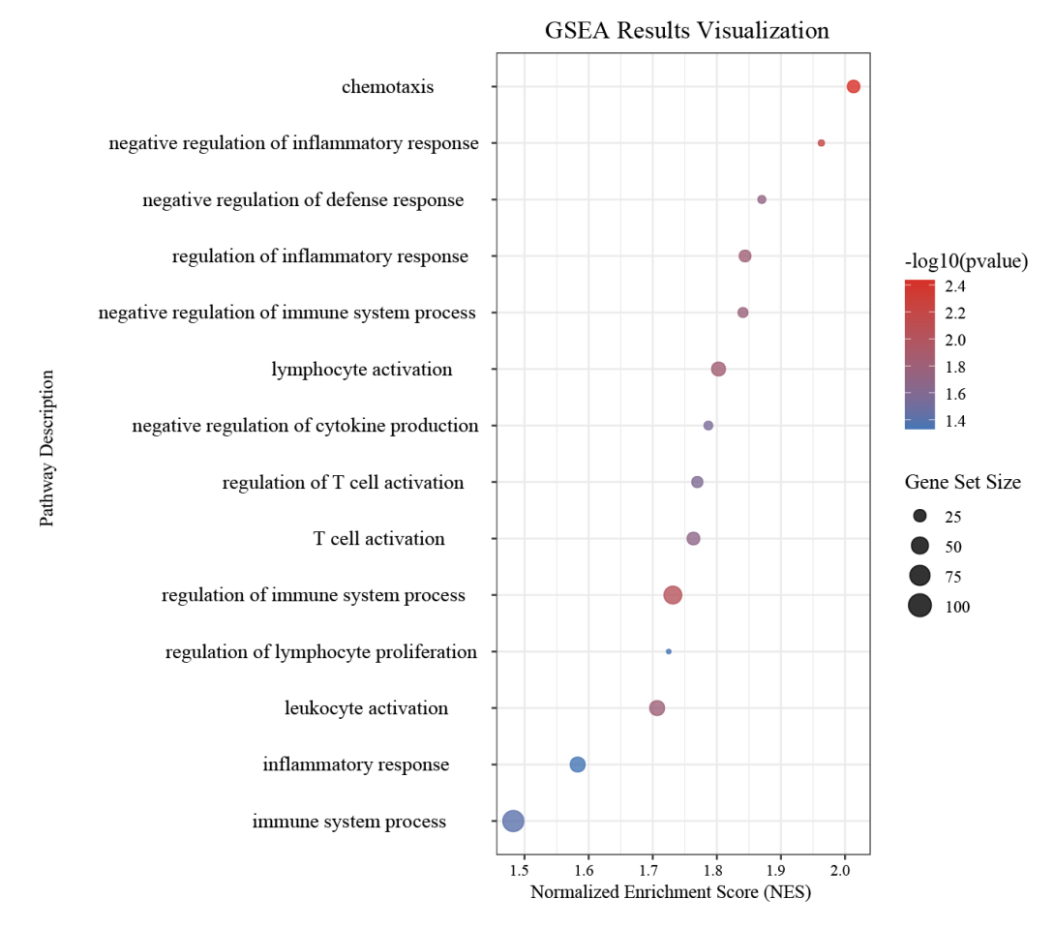


Fig S2. The dot plot presents the gene set enrichment analysis results of differentially expressed genes between the virus-infected and uninfected groups in the knockout strain HuBΔ*CD2v*Δ*A137R*.


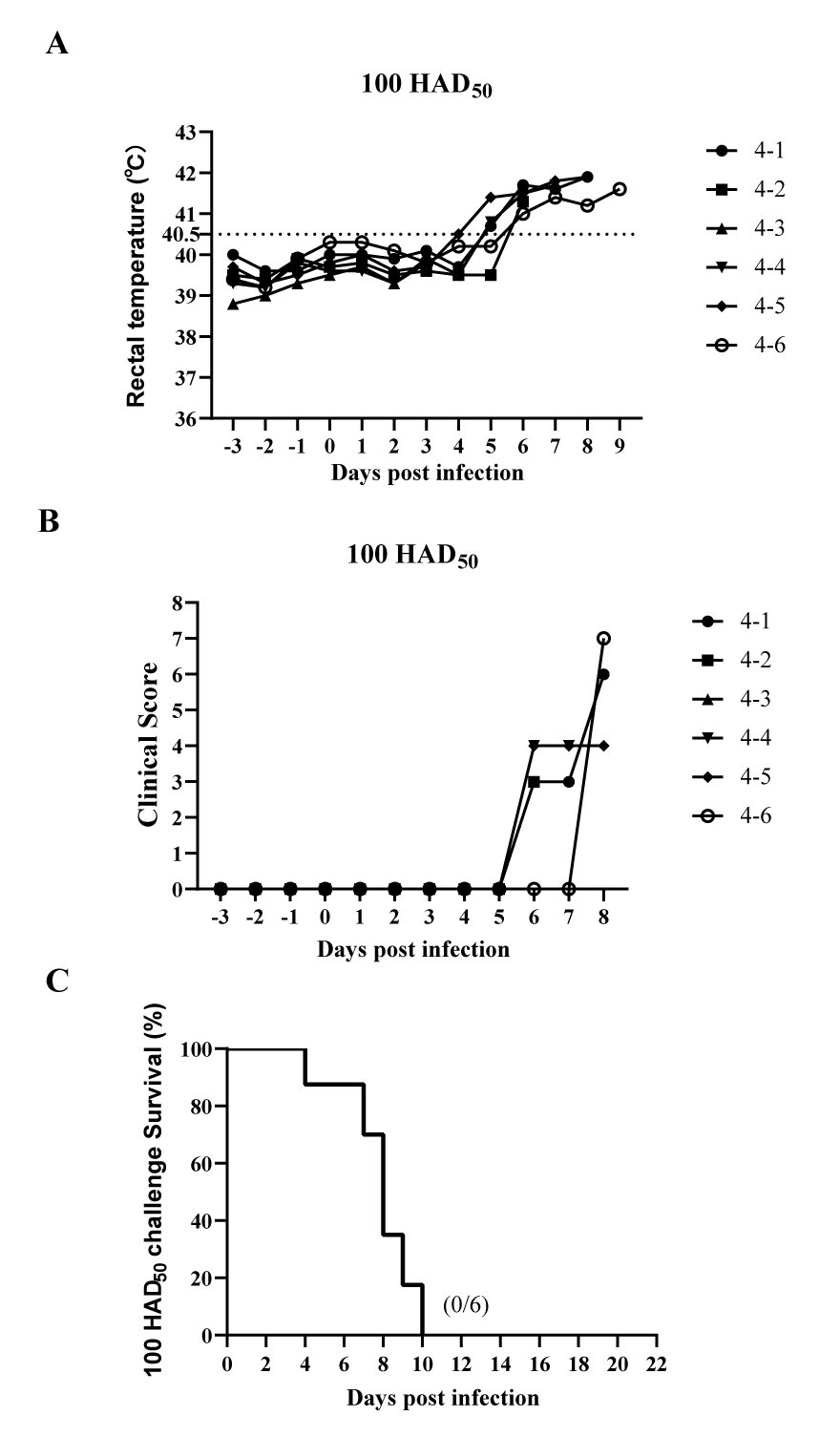


Fig S3. Piglets challenged by 100 HAD_50_ of the wildtype HuB/HH/2019. (A) The rectal temperature of all 6 piglets exceeded 40.5 ℃ for 3 days or more, and exceeded 41 ℃ at 6 dpi. (B) Clinical score and (C) Survival curve of the pigs challenged. 4-3 # died at 4 dpi without obvious symptoms, and the other piglets showed decreased appetite, depression, lethargy and shortness of breath at 6 dpi.4-2 #, 4-4 # and 4-5 # died at 7, 8 and 9 dpi, respectively. 4-1 # and 4-4 # showed depression, anorexia and euthanized at 8 and 9 dpi respectively.


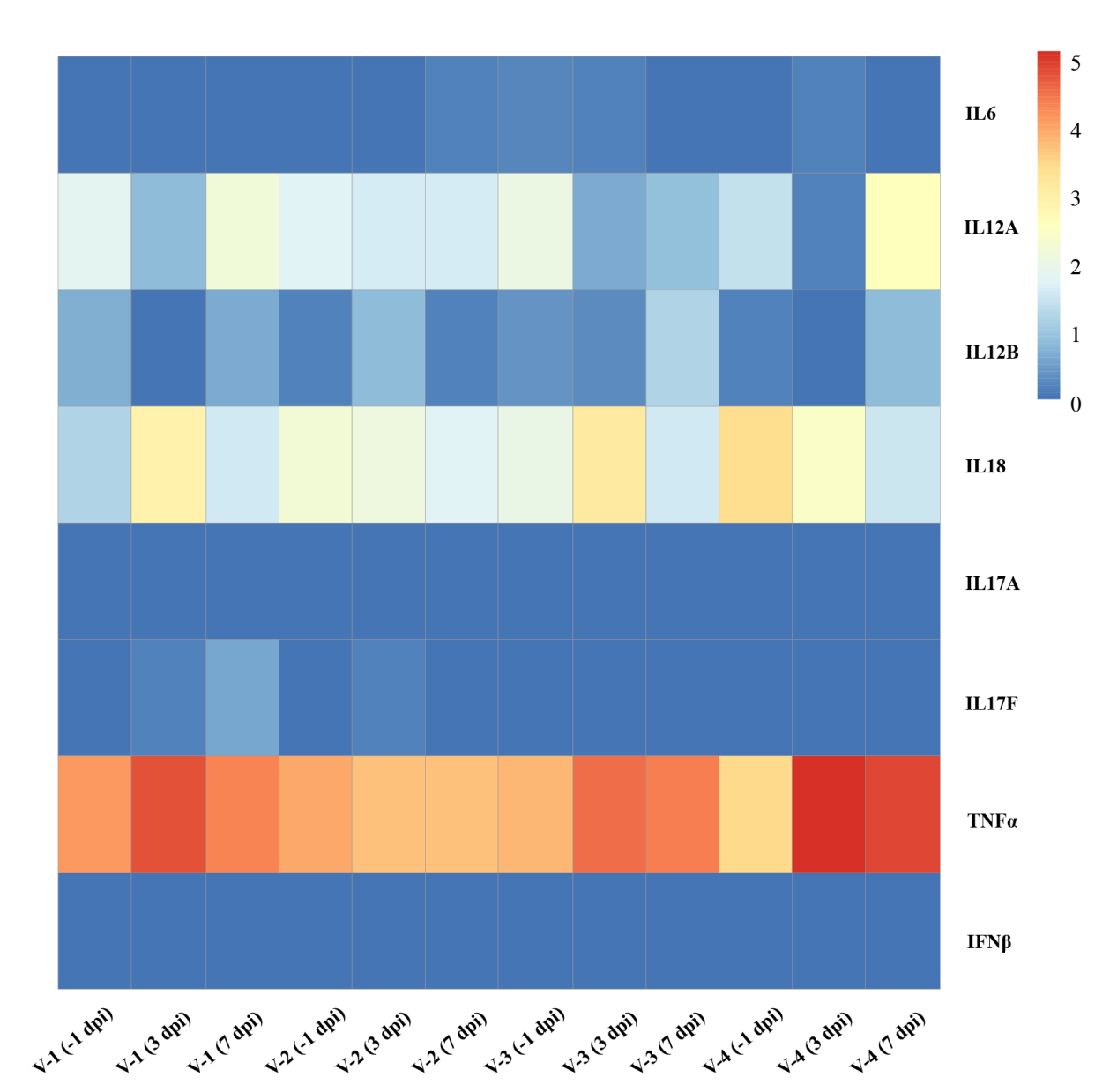


Fig S4. Heatmap of the expression of the key cytokines post-vaccination with HuBΔ*CD2v*Δ*A137R* at different dpi. Heatmap showing the expression of the key cytokines which were not significantly differentially expressed in host PBMC at different dpi. The color indicates the log2 (X+1)-transformed CPM of genes.

Table S1. Homology analysis of ASFV CD2v and A137R

| A137R  CD2v | 1 | 2 | 3 | 4 | 5 | 6 | 7 | 8 | 9 | 10 | 11 | 12 | 13 |  |
| --- | --- | --- | --- | --- | --- | --- | --- | --- | --- | --- | --- | --- | --- | --- |
| 1 |  | 100 | 100 | 100 | 100 | 100 | 100 | 100 | 99.3 | 99.3 | 99.3 | 84.7 | 84.7 | 1 HuB/HH/2019 Genotype Ⅱ |
| 2 | 100 |  | 100 | 100 | 100 | 100 | 100 | 100 | 99.3 | 99.3 | 99.3 | 84.7 | 84.7 | 2 Georgia 2007 NC_044959.2 Genotype Ⅱ |
| 3 | 100 | 100 |  | 100 | 100 | 100 | 100 | 100 | 99.3 | 99.3 | 99.3 | 84.7 | 84.7 | 3 HLJ2018 MK333180.1 Genotype Ⅱ |
| 4 | 100 | 100 | 100 |  | 100 | 100 | 100 | 100 | 99.3 | 99.3 | 99.3 | 84.7 | 84.7 | 4 Belgium Etalle wb 2018 MK543947.1 Genotype Ⅱ |
| 5 | 100 | 100 | 100 | 100 |  | 100 | 100 | 100 | 99.3 | 99.3 | 99.3 | 84.7 | 84.7 | 5 CzechRepublic 2017LR722600.1 Genotype Ⅱ |
| 6 | 100 | 100 | 100 | 100 | 100 |  | 100 | 100 | 99.3 | 99.3 | 99.3 | 84.7 | 84.7 | 6 GZ201801MT496893.1 Genotype Ⅱ |
| 7 | 100 | 100 | 1 00 | 100 | 100 | 100 |  | 100 | 99.3 | 99.3 | 99.3 | 84.7 | 84.7 | 7 SY18 MH766894.3 Genotype Ⅱ |
| 8 | 100 | 100 | 100 | 100 | 100 | 100 | 100 |  | 99.3 | 99.3 | 99.3 | 84.7 | 84.7 | 8 AnhuiXCGQ2018 MK128995.1 Genotype Ⅱ |
| 9 | 58.1 | 58.1 | 58.1 | 58.1 | 58.1 | 58.1 | 58.1 | 58.1 |  | 100 | 100 | 85.4 | 85.4 | 9 BA71V 2014 U18466.2 Genotype Ⅰ |
| 10 | 58.1 | 58.1 | 58.1 | 58.1 | 58.1 | 58.1 | 58.1 | 58.1 | 100 |  | 100 | 85.4 | 85.4 | 10 Bein97 AM712239.1 Genotype Ⅰ |
| 11 | 46.8 | 46.8 | 46.8 | 46.8 | 46.8 | 46.8 | 46.8 | 46.8 | 80.6 | 80.6 |  | 85.4 | 85.4 | 11 OUR T88-3 AM712240.1 Genotype Ⅰ |
| 12 | 64.4 | 64.4 | 64.4 | 64.4 | 64.4 | 64.4 | 64.4 | 64.4 | 54.9 | 54.9 | 44.5 |  | 100 | 12 Ken06.Bus KM111295.1 Genotype Ⅹ |
| 13 | 64.4 | 64.4 | 64.4 | 64.4 | 64.4 | 64.4 | 64.4 | 64.4 | 54.9 | 54.9 | 44.5 | 100 |  | 13 R35 MH025920.1 Genotype Ⅸ |

Table S2. RT-qPCR analysis of *p72*, *CD2v* and *A137R* genes in the HuB/HH/2019 and HuB△*CD2v*△*A137R* infected PAM cells. CT values from qPCR greater than 37 or undetectable were labeled as ''neg".

| Gene | HuB/HH/2019 | HuB△*CD2v*△*A137R* | Negative control |
| --- | --- | --- | --- |
| *p72* | 19.21 | 20.98 | neg |
| *CD2v* | 20.17 | neg | neg |
| *A137R* | 18.72 | neg | neg |

Table S3. Replication of ASFV in organs from the pig V-1# 27 days post HuB△*CD2v*△*A137R* inoculation. The copies/mL of *p72* detected by real-time qPCR were shown. CT values from qPCR greater than 37 or undetectable were labeled as ''neg".

| Pig No. | Heart | Liver | Lung | Spleen | Kidney | Duodenum | Tonsil | Submaxillary lymph nodes | Hepatogastric lymph node |
| --- | --- | --- | --- | --- | --- | --- | --- | --- | --- |
| V-1# | 2.4×10^4^ | neg | neg | neg | neg | neg | 5.4×10^4^ | 5.3×10^3^ | 1.6×10^4^ |

Table S4. gRNA sequences of *CD2v* and *A137R* genes of African swine fever strains. Nucleotide positions refer to the ASFV Pig HLJ 2018 genome sequence (GenBank accession number MK333180.1). PAM sequences are marked in bold.

| Name | Sequence | Position | Length |
| --- | --- | --- | --- |
| *CD2v-1* | ATGATATAAATGGAGTATCA**TGG** | 73503-73522 | 20 |
| *CD2v-2* | ACAATTATATGTGATATTTG**GGG** | 73740-73759 | 20 |
| *CD2v-3* | AAAGTATACTTGAATATAAC**TGG** | 73851-73870 | 20 |
| *A137R-1* | AGTTCTTACCAAACTCGACC**AGG** | 54579-54598 | 20 |
| *A137R-2* | TATAGGTGCATCGTTCCTCA**GGG** | 54684-54703 | 20 |
| *A137R-3* | CCAGGCGGTGTGGAATTCAG**GGG** | 54746-54765 | 20 |

Table S5. Primer and probe sequences used in this study.

| Target Gene | Name | Sequences(5’-3’) |
| --- | --- | --- |
| *P72* | P72-qf | GAACGTGAACCTTGCTA |
|  | P72-qr | GGAAATTCATTCACCAAATCC |
|  | P72-IN | FAM-TAAAGCTTGCATCGCA-MGB |
| *CD2v* | CD2v-qf | GACACCACTTCCATACATGAAC |
|  | CD2v-qr | GGACGCATGTAGTAAATAGGT |
|  | CD2v-IN | Cy5-CAGTCGTTATCAGTATAA-MGB |
| *A137R* | A137R-qf3 | CTTGAAATCCCTGAGGAACG |
|  | A137R qr3 | CGATGTCCCGAAATGAGTCT |
|  | A137R IN | ROX- CACCGCCTGGCATGA -MGB |
| *CD2v* | EP402R-f | AGTCGGGGCTACAATCCTTT |
|  | EP402R-r | AAGGGCTTTTGCAGATGAGA |
| *A137R* | A137R-f | TCCCAACATTAGTTGGAAACG |
|  | A137R-r | GAGACAAAGGCATCGTCCAC |

Table S6. Reference Clinical Score for Artificially Infected Pigs with ASFV

| **Item**  **Number** | **Observation**  **Item** | **Scoring Criteria** | **Score** |
| --- | --- | --- | --- |
| 1 | Mental State | Alert and able to stand up quickly | 0 |
|  |  | Slightly less active, can stand up slowly | 1 |
|  |  | Fatigued, can only stand up when forced and then lies down again | 2 |
|  |  | Lethargic, reluctant to stand | 3 |
| 2 | Body Tension | Relaxed, with a straight back | 0 |
|  |  | Muscles tense and back curved when standing | 1 |
|  |  | Back curved and walking stiffly | 2 |
|  |  | Whole - body convulsions | 3 |
| 3 | Body Condition | Well - rounded and plump | 0 |
|  |  | Abdomen appears slightly empty | 1 |
|  |  | Abdomen is empty - looking and slightly emaciated | 2 |
|  |  | Emaciated, with visible bones | 3 |
| 4 | Respiratory Condition | Respiratory rate is 10 - 15 breaths per minute, no abdominal breathing | 0 |
|  |  | Respiratory rate > 20 breaths per minute | 1 |
|  |  | Respiratory rate > 20 breaths per minute with obvious abdominal breathing | 2 |
|  |  | Respiratory rate > 30 breaths per minute, gasping with an open mouth | 3 |
| 5 | Locomotion | Moves freely | 0 |
|  |  | Walks slowly, adjusts posture slowly | 1 |
|  |  | Visible ataxia, walks unsteadily | 2 |
|  |  | Completely uncoordinated, unable to walk | 3 |
| 6 | Skin Condition (mainly ears, nose, legs, tail) | Skin is pink and smooth | 0 |
|  |  | Localized skin redness | 1 |
|  |  | Localized skin turns purple and cool, with a few ecchymoses | 2 |
|  |  | Skin is dark red, dull, and mostly bleeding | 3 |
| 7 | Eyes/Conjunctiva | Sharp vision, pink and moist conjunctiva | 0 |
|  |  | Conjunctiva turns red, with clear secretions | 1 |
|  |  | High - level inflammation, with turbid secretions | 2 |
|  |  | High - level inflammation, with purulent secretions | 3 |
| 8 | Appetite | Eager to eat, shows signs of hunger | 0 |
|  |  | Eats slowly | 1 |
|  |  | Loss of appetite | 2 |
|  |  | Complete lack of appetite | 3 |
| 9 | Fecal Condition | Soft and of appropriate amount | 0 |
|  |  | Dry and decreased fecal output | 1 |
|  |  | Small amount of dry, bloody feces, or diarrhea | 2 |
|  |  | No feces, with rectal mucus secretion, or bloody diarrhea | 3 |
| 10 | Feed Utilization | Feeding trough is emptied and cleaned | 0 |
|  |  | Almost no feed left in the trough | 1 |
|  |  | Only part of the feed is consumed | 2 |
|  |  | Feed remains untouched | 3 |
